# Epirubicin-sensitive detection with a CoWO_4_/reduced graphene oxide modified screen-printed electrode

**DOI:** 10.5599/admet.2733

**Published:** 2025-05-08

**Authors:** Somayeh Tajik, Razieh Moghimian, Hadi Beitollahi

**Affiliations:** 1 Research Centre of Tropical and Infectious Diseases, Kerman University of Medical Sciences, Kerman, Iran; 2 Environment Department, Institute of Science and High Technology and Environmental Sciences, Graduate University of Advanced Technology, Kerman, Iran

**Keywords:** CoWO_4_/reduced graphene oxide nanocomposite, chemically modified electrode, urine sample, cancer, drug analysis

## Abstract

**Background and purpose:**

Because of its antimetabolic and cytotoxic qualities, epirubicin (EP), a crucial chemotherapeutic drug, has been used to treat a number of cancers, including those of the prostate, breast, ovary, stomach, lung, and colon.

**Experimental approach:**

In this study, CoWO_4_/reduced graphene oxide (CoWO_4_/rGO) nanocomposite was synthesised and characterised by field emission-scanning electron microscopy and energy-dispersive X-ray spectroscopy. To provide a novel sensing platform for EP determination, a screen-printed electrode (SPE) surface was modified using the as-fabricated CoWO_4_/rGO nanocomposite.

**Key results:**

Using voltammetric techniques, the electrochemical behaviour of the CoWO_4_/rGO nanocomposite modified SPE (CoWO_4_/rGO/SPE) for the EP detection was examined. CoWO_4_/rGO significantly reduced the overpotential of the EP redox reaction and increased the rate of electron transfer between the electrode and analyte as compared to bare SPE. EP was quantitatively analysed using differential pulse voltammetry.

**Conclusions:**

It was discovered that the linearity range was 0.01 to 190.0 μM. The sensitivity and limit of detection were 0.1529 μA μM^-1^ and 0.007 μM, respectively. Additionally, the constructed CoWO_4_/rGO/SPE sensor's practical applicability was investigated in pharmaceutical samples with good recovery outcomes.

## Introduction

One of the anthracycline derivatives is epirubicin (EP). Because of its cytotoxic and antimetabolic qualities, EP has been used as a key chemotherapeutic drug to treat breast, prostate, ovarian, gastric, lung, and colorectal cancers, among other cancers. In patients with prostate cancer, EP has continuously shown cytotoxic effects, whether given as a monotherapy or in combination with other treatment drugs [1-5]. It is still unclear exactly what method EP works via. It has been suggested that the chemical mainly targets the fast DNA replication in cancerous cells. The steric changes that are incorporated into the structure of EP affect the stability of the DNA-anthracycline complex, which causes the analogue to enter and exit the tumour and normal cells more quickly (which breaks the helical structure of DNA), which in turn prevents the synthesis and replication of DNA and RNA and tumour cell growth [6-11]. The evaluation of EP concentrations in human biological fluids is helpful in adjusting pharmacological dosages in the treatment strategy that targets cancer cells because of the potentially dangerous nature of high medication dosages for patients. The measurement of EP in genuine samples has been accomplished using a variety of approaches and quantitative analytical techniques, such as spectrophotometric techniques, electrophoresis, and liquid chromatography (LC) [12-19]. Trace quantities of EP in pharmaceutical formulations and biological specimens have been widely detected using electrochemical techniques, which are known for their ease of use, sensitivity, and affordability [20-22].

With its small shape and ability to link to portable equipment for on-site analyte detection, screen-printed electrodes (SPEs) made using microfabrication technology have three electrodes printed on a single strip. These devices may also be surface-modified with different compositions and have a versatile design. SPEs support green chemical concepts, such as the creation of safe products, and are affordable, simple to manufacture, and appropriate for mass production. Furthermore, SPEs provide great sensitivity, low energy consumption, linear response, and the ability to operate efficiently at room temperature [23-26].

Unmodified electrodes often have low sensitivity and a high over-potential, which causes surface fouling to build up gradually over time. The electrode surface must be modified for the electrochemical detection of different analytes. Enhancing the electron exchange between the electrode surface and the electro-active species is the primary objective of electrode modification. Consequently, several investigations have been carried out to create modified electrodes utilizing a range of substances and nanostructures [27-32]. High specific surface area, exceptional conductivity, a large number of surface-active sites, and potent catalytic activity are some benefits of nanomaterials. These features can significantly increase the stability and sensitivity of sensors. Additionally, nanomaterials can facilitate electrochemical processes and improve electron transfer efficiency by acting as catalysts. Carbon nanostructures have many structural and property differences, including graphene oxide (GO) and its derivatives, which lead to various uses. Since its discovery, graphene, one of the carbon allotropes, has made tremendous strides in studying carbon nanostructures. A two-dimensional honeycomb lattice of carbon atoms is used to form single-layer graphene sheets. GO's huge surface area and electrical conductivity, among other qualities, make it a perfect and valuable material for various electrochemical applications, such as energy conversion, sensing, and storage [33-37].

Because of the synergistic effects between two transition metals, binary transition metal oxides (BTMOs) have outstanding electrochemical characteristics, making them effective and appropriate electrode materials. CoWO_4_ stands out as a noteworthy molecule with potent chemical and catalytic capabilities. Many attempts have been made to improve BTMOs' electrochemical performance. One efficient strategy is developing a technique for combining highly conductive carbon nanostructures with binary transition metal oxides to create a nanocomposite [37-45]. Because of their high electrical conductivity, large surface area, and advantageous mechanical properties, carbon nanostructures, like two-dimensional reduced graphene oxide (rGO), can be integrated with BTMOs to greatly enhance the electrical conductivity and electrochemical properties of the resulting nanocomposites [46,47].

Using a CoWO_4_/rGO nanocomposite, this work offers a simple and sensitive electrochemical sensing platform for improved EP detection. Due to its greater electrical conductivity and bigger active surface area, the CoWO_4_/rGO/SPE sensing platform demonstrated better electrochemical performance for EP detection than unmodified SPE. With a low limit of detection (LOD) and good sensitivity over a broad linear detection range, quantitative studies showed that the proposed sensor had exceptional electrochemical sensing capabilities for EP determination. Additionally, examining the injection sample showed how well the developed sensor worked. This work's main novelty is the effective modification of SPE in the voltammetric determination of EP using the CoWO_4_/rGO nanocomposite, which has beneficial features.

## Experimental

### Apparatus and chemicals

Electrochemical measurements are conducted using an Autolab potentiostat/galvanostat. A screen-printed electrode (SPE) from DropSens (DRP-110, Spain) is utilized, incorporating three standard electrodes: a silver pseudo-reference electrode, a graphite counter electrode, and a graphite working electrode. pH measurements are performed using a Metrohm 710 pH meter. All other reagents, including epirubicin, were of analytical grade and sourced from Merck. Buffer solutions were prepared with orthophosphoric acid and its corresponding salts, covering a pH range of 2.0 to 9.0.

### Synthesis of CoWO_4_/rGO nanocomposite

**Figure 1.**
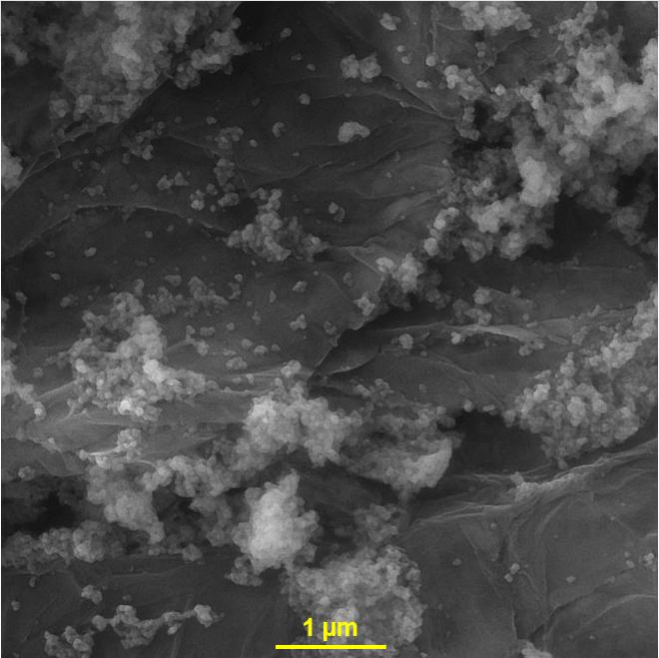
FE-SEM image of CoWO_4_/rGO nanocomposite

With a few adjustments, the CoWO_4_/rGO nanocomposite's synthesis was carried out using the methodology described by Xu *et al*. [48]. To do this, 60 mg of GO was dissolved in 40 mL of deionised water, and the mixture was ultrasonically agitated for one hour to create an aqueous suspension of GO. Following ultrasonication, the aforementioned suspension was mixed with aqueous solutions (10 mL containing 2 mmol CoCl_2_.6H_2_O (0.475 g) and 10 mL containing 2 mmol Na_2_WO_4_.2H_2_O (0.659 g)) and magnetically swirled for one hour. The GO suspension with metal salts was then put into a 100 mL stainless-steel autoclave lined with Teflon. It was then baked for 12 hours at 180 °C before cooling naturally to room temperature. The product, which was produced after washing and drying at 70 °C for 15 hours, was identified as the CoWO_4_/rGO nanocomposite after the prepared precipitate was collected by centrifugation. Figure 1 shows an atypical FE-SEM.

**Figure 2.**
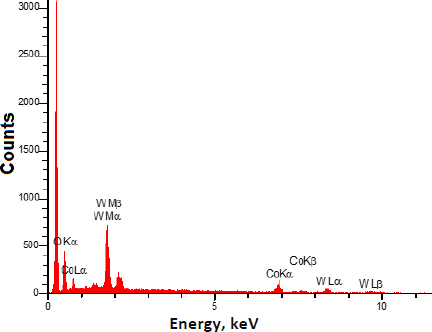
EDX spectrum of CoWO_4_/rGO nanocomposite

Additionally, the EDX analysis (Figure 2) shows that the prepared nanocomposite contains Co, W, C, and O elements without any impurities.

### Preparation of the electrode

A CoWO4/rGO nanocomposite is added to the screen-printed working electrode using a straightforward drop-casting technique. After 30 minutes of ultrasonication, the CoWO_4_/reduced graphene oxide nanocomposite (1 mg) was dispersed in 1 millilitre of the aqueous solution to create the stock solution. The screen-printed working electrode surface is then covered with a 5 μl CoWO4/rGO nanocomposite suspension. After that, the solvent was allowed to evaporate at room temperature.

### Preparation of real samples

Immediately after collection, the drug-free human urine specimens are kept in a refrigerator. First, 10 ml of each sample was utilised and centrifuged for 600 s at 2000 rpm. A 0.45 μm filter was used to filter the supernatant. After that, different amounts of the treated urine samples were collected, put in a flask, and diluted using a pH 7.0 phosphate buffer solution (PBS). Different amounts of epirubicin were added to these samples. The concentrations of epirubicin are measured using the standard addition method.

10.0 mL of PBS (0.1 M) at pH 7.0 was combined with 1.0 mL of an epirubicin ampoule that contained 2 mg in 1 mL. To attain the calibration mark, various amounts of the resultant diluted solution were poured into a series of 25 mL volumetric flasks and further diluted with PBS solution. The standard addition method was used to conduct the study.

## Results and discussion

### Electrochemical behaviour of EP on the various electrodes

**Figure 3.**
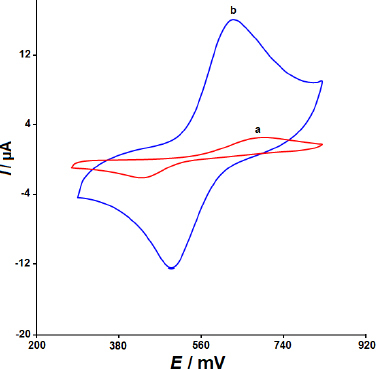
The cyclic voltammograms of (a) bare SPE and (b) CoWO_4_/rGO/SPE at a scan rate of 50 mVs^-1^ with 100.0 μM epirubicin in 0.1 M PBS at pH 7

In the pH range of 2.0 to 9.0, the effect of pH on the present responsiveness of CoWO_4_/rGO/SPE towards EP oxidation was investigated. The findings showed that at a pH of 7.0, the current response of CoWO_4_/rGO/SPE to EP oxidation peaked. Therefore, pH 7.0 was chosen for more research and analysis. To assess the CoWO_4_/rGO nanocomposite's performance in the electrochemical measurement of EP, cyclic voltammetry tests was conducted (Figure 3). The CV responses of the CoWO_4_/rGO/SPE (curve b) and an unmodified SPE (curve a) were measured in 0.1 M PBS at pH 7.0 with 100.0 μM EP. As can be observed, both the modified and unmodified electrodes displayed distinct anodic/cathodic redox peaks for EP. On the CoWO_4_/rGO/SPE, however, a significant impact on EP detection was noted. For the redox reaction of EP, the unmodified SPE showed a poor voltammetric response, resulting in comparatively lower current values. Compared to bare electrodes, the CoWO_4_/rGO/SPE showed a stronger voltammetric response to EP. Furthermore, in contrast to the unmodified SPE, the redox peaks of EP were observed at lower potentials in the CoWO_4_/rGO/SPE. The beneficial benefits of the CoWO_4_/rGO nanocomposite and their synergistic effects may be the reason for the CoWO_4_/rGO/SPE's higher sensitivity to EP as compared to bare SPE, according to the comparison of these CVs.

### Effect of scan rate

**Figure 4.**
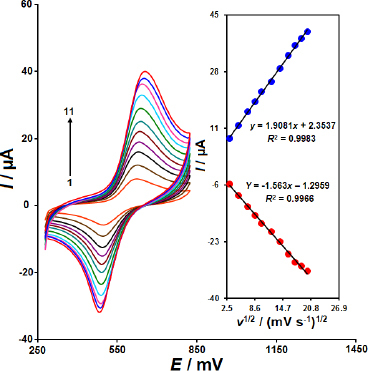
Cyclic voltammograms of 100.0 μM EP at the CoWO_4_/rGO/SPE surface in 0.1 M PBS (pH 7.0) at various scan rates (1-11 respectively, corresponding to 10, 25, 50, 75, 100, 150, 200, 250, 300, 350 and 400 mV s^-1^). Inset: redox peak currents plotted linearly against ν^1/2^

To examine the impact of scan rates between 10 and 400 mV s^-1^; on the redox peak currents (*I*_pa_, *I*_pc_) and peak potential, CVs of 100.0 μM EP were recorded using the CoWO_4_/rGO/SPE sensor at varying scan rates (Figure 4).

As the scan rate increases, the peak current magnitude rises accordingly. Simultaneously, the oxidation peak potential of EP shifts toward more positive values, while the reduction peak potential moves toward more negative values. The inset of Figure 4 shows a linear relationship between redox peak currents and the square root of the scan rate (ν^1/2^) for EP. This result demonstrates that the CoWO_4_/rGO/SPE surface is diffusion-controlled for the redox process of EP.

### Chronoamperometric studies

**Figure 5.**
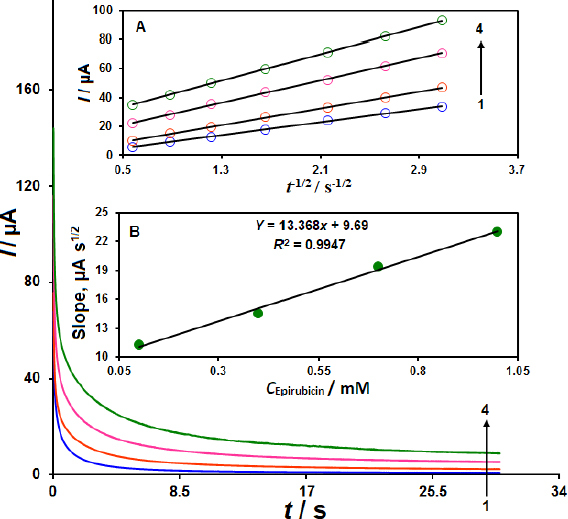
Chronoamperograms of EP at different concentrations (0.1 to 1.0 mM) acquired at CoWO_4_/rGO/SPE in PBS (0.1 M with pH 7.0). The concentrations of EP represented by the numbers 1 to 4 are 0.1, 0.4, 0.7 and 1.0 mM. *I* vs. *t*^-1/2^ linear graphs obtained from chronoamprograms are shown in Inset A, and the slopes of these lines are shown against *C*_EP_ in Inset B

The chronoamperometric evaluations of different EP concentrations performed at the CoWO_4_/rGO/SPE are shown in Figure 5. This method makes it possible to calculate the diffusion coefficient (*D)* of EP. For an electro-active species with a diffusion coefficient (*D* / cm^2^ s^-1^), the current of an electrochemical reaction is described by the Cottrell equation (*I* = *nFAC*_b_*D*^1/2^π^-1/2^*t*^-1/2^). The best-fit curves were used for the different EP levels (Inset A (Figure 5). The slope of the derived linear equations was then displayed against the EP levels (Inset B, Figure 4). The *D* parameter for EP was determined to be 1.5p10^-5^ cm^2^ s^- 1^ using the Cottrell equation to the slopes found in the experimental plots.

### Voltammetric detection of EP on CoWO_4_/rGO/SPE

**Figure 6.**
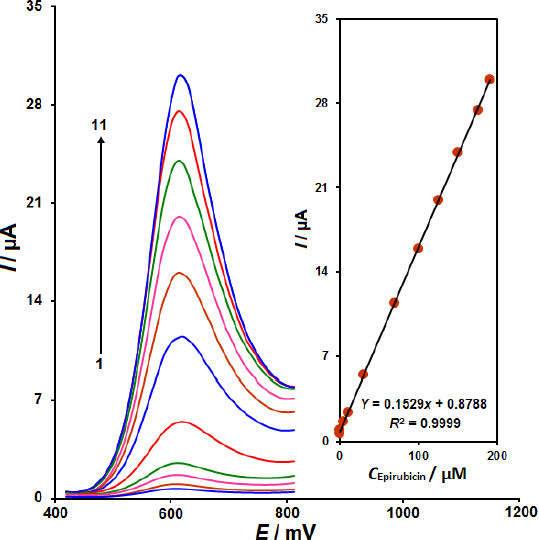
The recorded DPVs for the CoWO_4_/rGO/SPE in the 0.1 M PBS at pH 7.0 containing EP at different concentrations (voltammograms 1 to 11 related to 0.01, 0.1, 5.0, 10.0, 30.0, 70.0, 100.0, 125.0, 150.0, 175.0 and 190.0 μM, respectively). Inset: Corresponding calibration plot of *I*_pa_ *vs.* concentrations of EP

The main goal of this analysis is to develop a sensing electrode for the detection of low EP concentrations using a CoWO_4_/rGO nanocomposite. Thus, DPV was used to study the oxidation of EP at CoWO_4_/rGO/SPE in 0.1 M PBS (pH 7.0) by adjusting its concentration between 0.01 and 190.0 μM under the ideal circumstances of 50 mV s^-1^ scan rate, 0.01 V step potential, and 0.025 V pulse amplitude (Figure 6). A plot of *I*_pa_ against EP concentration is shown in Figure 6 (Inset), which shows a linear connection between *I*_pa_ and EP concentration. As the concentration of EP rises, so does the peak current. The linearity is obtained in the range of 0.01 and 190.0 μM. Additionally, 0.007 μM was determined to be the LOD value.

### Repeatability and selectivity studies

The DPV responses at CoWO_4_/rGO/SPE in PBS 0.1 M (pH 7.0) towards 70.0 μM EP were recorded 15 times to determine the devised approach's repeatability. The modified SPE preserved 98.8 % of its current response from the initial test, indicating strong repeatability, according to measurements conducted under identical circumstances.

The DPV measurements were carried out in 0.1 M PBS with 70.0 μM EP in the presence of several interferents to evaluate the selectivity of the CoWO_4_/rGO/SPE sensor. The results revealed that the current response of EP was not significantly affected by glucose, dopamine, L-cysteine, glycine, and tryptophan, Na^+^, K^+^, Br^-^ and NO_3_^-^ (the signal change was less than ±5 %).

### Practical application CoWO_4_/rGO/SPE sensor in real specimens

The CoWO_4_/rGO/SPE was utilised to assess EP in urine samples and injection using the standard addition method to demonstrate the created sensor's applicability. The findings of this study are displayed in Table 1. The results showed that recoveries that ranged from 97.3 to 104.4 % were satisfactory. Furthermore, the designed sensor's excellent accuracy was demonstrated by the obtained RSDs (*n* = 5) being less than 3.6%. Thus, it is possible to analyse EP in actual specimens using the CoWO_4_/rGO/SPE sensor.

**Table 1.** Utilizing the CoWO_4_/rGO/SPE sensor to measure EP in actual samples

Sample	Added concentration, μM	Found concentration, μM	Recovery, %	RSD, %
EP injection	0	4.1	-	3.4
	1.0	5.0	98.0	2.1
	3.0	7.3	102.8	1.8
	5.0	9.5	104.4	2.9
	7.0	11.0	99.1	3.0
Urine	0	-	-	-
	5.0	5.1	102.0	1.8
	7.5	7.3	97.3	3.6
	10.0	10.4	104.0	2.2
	12.5	12.4	99.2	2.7

## Conclusion

The CoWO_4_/rGO nanocomposite was created using a straightforward process and examined using the FE-SEM and EDX methods. EP was determined voltammetrically using CoWO_4_/rGO/SPE. Excellent EP detection capabilities are demonstrated by the CoWO_4_/rGO/SPE due to the synergy between CoWO_4_ and rGO. With a low limit of detection of 0.007 μM, the developed sensor displayed linear dynamic ranges in the range of 0.01-190.0 μM of EP concentration. To sum up, the suggested sensor has been shown to be a reliable instrument for precisely detecting EP in actual specimens, producing positive outcomes.
